# From Epidemiology to Daily Life: Linking Daily Life Stress Reactivity to Persistence of Psychotic Experiences in a Longitudinal General Population Study

**DOI:** 10.1371/journal.pone.0062688

**Published:** 2013-04-23

**Authors:** Dina Collip, Johanna T. W. Wigman, Inez Myin-Germeys, Nele Jacobs, Catherine Derom, Evert Thiery, Marieke Wichers, Jim van Os

**Affiliations:** 1 Department of Psychiatry and Psychology, School of Mental Health and Neuroscience, Maastricht University Medical Centre, Maastricht, The Netherlands; 2 Faculty of Psychology, Open University of The Netherlands, Heerlen, The Netherlands; 3 Department of Human Genetics, University Hospital Gasthuisberg, Catholic University Leuven, Leuven, Belgium; 4 Department of Neurology, Ghent University Hospital, Ghent, Belgium; 5 King's College London, King's Health Partners, Department of Psychosis Studies, Institute of Psychiatry, De Crespigny Park, London, United Kingdom; University of Cardiff, United Kingdom

## Abstract

Subclinical psychotic experiences at the level of the general population are common, forming an extended psychosis phenotype with clinical psychosis. Persistence of subclinical experiences is associated with transition to later mental disorder. Increased daily life stress reactivity is considered an endophenotype for psychotic disorders. We examined, in a longitudinal framework, whether baseline momentary assessment markers of stress reactivity would predict persistence of subclinical psychotic experiences over time. In a general population sample of female twins (N = 566), the Experience Sampling Method (ESM; repetitive random sampling of momentary emotions, psychotic experiences and context) was used to assess (emotional and psychotic) daily life stress reactivity. Persistence of subclinical psychotic experiences was based on the Community Assessment of Psychic Experiences (CAPE), assessed three times over 14 months post-baseline. It was investigated whether baseline daily life emotional and psychotic stress reactivity predicted persistence of psychotic experiences over time. Higher levels of emotional stress reactivity (a decrease in positive and an increase in negative affect in response to stress), and increased psychotic reactivity to daily stress was found in individuals with persistent psychotic experiences over time compared to individuals with transient psychotic experiences. The results suggest that markers of daily life stress reactivity may predict “macro-level” persistence of normally transient expression of psychotic liability over time. Linking daily life markers of altered reactivity in terms of emotions and psychotic experiences to longitudinal persistence of psychotic experiences, associated with increased risk of transition to overt mental disorder, may contribute to earlier and more accurate diagnosis of risk.

## Introduction

Psychiatric research is complementing its previous focus on categorical, heterogeneous disease entities with dimensional approaches towards psychopathology. For example, there is evidence that subclinical psychotic experiences at the level of the general population are common, and represent an extended psychosis phenotype outside the boundaries of clinical disorder [1]. Psychotic experiences may be considered truly dimensional, extending across most mental disorders including common mental disorder [2], in which they impact negatively on course and outcome, as well as occasioning a more “schizophrenia-like” risk factor profile [3]. In fact, the majority of individuals who report psychotic experiences present with other mental disorders, particularly depression or anxiety disorders [3], [4]. In the general population, psychotic experiences predict later psychotic and, to a lesser extent, affective disorders [5], even when not considered clinically relevant [6] and particularly when persistent [7], [8].

High levels and persistence of psychotic experiences is influenced by both environmental and genetic factors [9], [10]. One hypothesis is that persistence of subclinical psychotic experiences reflects the behavioral expression of stress sensitization [11], [12]. Sensitization refers to the phenomenon that the response to an environmental risk factor increases in intensity with repeated exposure to this risk factor. Eventually, this may lead to a lasting change in response amplitude [11], [12]. It is hypothesized that indicators of early behavioral sensitization precedes persistence of psychotic symptoms.

Earlier work has shown that markers of (behavioral) sensitization can be identified at the level of everyday life experience. For example, in patients with clinical psychotic disorder [13] and in their siblings [14], increased stress reactivity, in the form of both emotional and psychotic reactivity to daily hassles, has been reported. Given that daily life stress reactivity is considered a marker of sensitization, and that the process of sensitization is thought to mediate the expression of psychosis, it is attractive to hypothesize, in a longitudinal framework, that baseline daily reactivity to stress (assessed with momentary assessment technology in daily life) predicts persistence of subclinical psychotic experiences, which can be considered a measure of psychometric psychosis risk. Linking daily life markers of stress reactivity to longitudinal persistence of psychotic experiences and increased risk of transition to overt mental disorder allows for earlier and more accurate diagnosis of risk and might moreover have important implications for (very)early intervention and risk reduction [15].

In a longitudinal study of young adult female twins from the general population, we examined the following questions:

- Do individuals with stable high levels (i.e. persistence) of subclinical psychotic experiences over time (macro-level) at baseline show increased emotional stress reactivity in daily life (micro-level) compared to those with stable low levels of psychosis?
- Do individuals with stable high levels (i.e. persistence) of subclinical psychotic experiences over time (macro-level) at baseline show increased psychotic stress reactivity in daily life (micro-level) compared to those with stable low levels of psychosis?

We hypothesized that stable high levels (i.e. persistence) of subclinical psychotic experiences would be associated with increased emotional and psychotic reactivity to stress in daily life at baseline.

## Methods

### Sample

This study forms part of a general population twin study that investigates gene – environment interactions in vulnerability for mental disorders, as described previously [16], [17]. Given that genetic effects on psychopathology are likely sex-specific to a degree, only women were included in the original study, meaning that the current analyses pertain to women only. Participants (twins) were recruited from the East-Flanders Prospective Twin Survey, a population-based survey that has prospectively recorded all multiple births in the province of East Flanders since 1964 [16], [17]. Originally, the sample included 621 subjects (575 twins and 46 of their non-twin sisters). The 46 non-twin sisters were excluded, as well as three subjects with missing zygosity and six subjects who participated without their twin. Thirty-seven individuals did not participate in the ESM study or were excluded because they had missing or too few valid ESM self reports. The final sample thus consisted of 529 subjects (323 individuals were monozygotic, and 206 were dizygotic twins), with mean age 27.2 years (SD 7.4; range 18–46). Participants were interviewed five times (T0–T4) at approximately 3- to 4-monthly intervals. Participants were white and of Belgian origin. Sixty-two percent had a higher education, 36% had followed higher secondary school, and 2% had finished primary school only and the majority was employed (62%) and in a relationship (76%). There were five measurement points, including a baseline (T0) and four follow-up measurements (T1–T4). The average number of days between T0 and T1 was 132, 91 between T1 and T2, 116 between T2 and T3, and 91 between T3 and T4. Baseline measurements were carried out at individuals' homes, and follow-up measurements were collected using questionnaires and telephone interviews. All interviews were performed by trained research psychologists or graduate psychological assistants. The study was approved by the ethics committee of the Maastricht University Medical Centre and all participants provided written informed consent after receiving complete description of the study.

### Instruments

The positive item scale of the Community Assessment of Psychic Experiences (CAPE) (20 self-reported items) was used to assess subclinical psychotic experiences [18]. The CAPE is based on the Peters Delusions Inventory (PDI), modified to also include hallucinatory experiences [19]. Each item in the CAPE rates two aspects of subclinical psychotic experiences: (i) frequency and (ii) associated distress, both rated on a four-point scale of never/not distressed (1); sometimes/a bit distressed (2); often/quite distressed (3); nearly always/very distressed (4). The CAPE was assessed at T0, T2, and T4. The frequency items showed good internal consistency (Cronbach's alpha >0.96 at all three measurements). Standardized sum scores of the positive items subscale were used as indicators for the growth model.

The Structured Clinical Interview for DSM-IV Axis I disorders (SCID) was administered at T0 (and T4). To measure baseline psychotic symptoms, the sum score of the subscales delusions (consisting of 15 items) and hallucinations (consisting of eight items) of the SCID was used.

### Trajectories

In a previous paper, growth mixture modeling was used to identify two different developmental trajectories of the CAPE items, assessed at the three time points of the Flanders twin study [20]. These developmental trajectories represent different courses of subclinical psychotic experiences over time. Two differential subgroups were found: a larger group (N = 467; 88% of the current sample; in original sample N = 496) with a persistently low (subclinical) expression of psychosis (referred to as Low group) and a smaller group (N = 62; 12% of the current sample; in original sample N = 70) with a persistently high (subclinical) expression of psychosis (referred to as Persistent group). These two groups can be interpreted as representing different levels of vulnerability for psychosis, since they were differentially associated with psychopathology, risk factors for psychosis and functioning (for more details see Wigman et al., 2011) [20].

### Experience Sampling Method (ESM)

The Experience Sampling Method (ESM) is a random time-sampling self-assessment technique that has been shown to be feasible, valid, and reliable in general and patient populations [21], [22]. ESM data were collected at baseline (T0). Subjects received a digital wristwatch that emitted a signal ten times a day on five consecutive days, at unpredictable moments between 7:30 a.m. and 10:30 p.m. After each ‘beep’, subjects completed ESM self-assessment forms concerning current context, thoughts, emotions, and psychotic experiences. Subjects were instructed to complete their reports immediately after the beep, thus minimizing memory distortions. Reports were considered valid when subjects responded within 15 minutes after the beep, as determined by comparing the actual beep time with the reported time of completion. For inclusion in the analyses, participants had to have provided valid responses to at least one-third of the emitted beeps [23].

### ESM measures

#### Event stress

Event stress was conceptualized in terms of subjective appraisals of events and minor disturbances that continually occur in the natural flow of daily life. After each beep, participants were asked to report the most important event that had happened between the current and the previous report and then to rate this event on a 7-point, bipolar Likert scale (−3 =  *very unpleasant*, 0 =  *neutral*, 3 =  *very pleasant*). For the current analyses, all positive responses were recoded as 0, and the negative responses were recoded so that high scores reflect more unpleasant and potentially stressful events (0 =  *neutral*, 3 =  *very unpleasant*) [24].

#### Activity stress

For activity-related stress, participants rated their current activity on three self-report items scored on 7 point Likert scales, namely “I am not skilled to do this activity”, “I would rather do something else” and “This activity requires effort”. The mean of these items represented the activity-related stress scale (alpha = .50).

#### Social stress

Social stress was measured by asking subjects whether they were alone at the time of the beep. If not alone, they were asked whether they liked the company they were in at that moment. This was rated on a 7-point Likert scale. The scale was reversed so that higher scores represent higher disliking of being in that company.

#### Paranoid ideation

Paranoid ideation was assessed with the ESM item *“I feel suspicious”* rated on a 7-point Likert scales *(*1 =  *not at all* to 7 =  *very)* [25].

#### Negative affect

Negative affect (NA) was assessed as the mean score on 5 ESM items, rated on 7-point Likert scales *(*1 =  *not at all* to 7 =  *very): “I feel insecure”, “I feel lonely”, “I feel anxious”, “I feel down” and “I feel guilty” (alpha =  0.73)* [26].

#### Positive affect

Positive affect (PA) was assessed as the mean score on 4 ESM items, rated on 7-point Likert scales *(*1 =  *not at all* to 7 =  *very): “I feel happy”, “I feel cheerful”, “I feel relaxed” and “I feel satisfied” (alpha = 0.86)* [26].

### Statistical analyses

#### Main Analyses

Momentary ESM data were analysed using multilevel linear regression techniques, which take the hierarchical structure of the data (repeated measurements clustered in persons) into account. In addition, the sample consisted of twin pairs, resulting in a further level of clustering. Thus, in the current study, repeated momentary measurements (level 1) were clustered within subjects (level 2), some of whom were members of the same twin pair (level 3). Data were analyzed using the XTMIXED multilevel random regression routine in STATA 11.0 [27], providing non-standardized regression coefficients of the predictors in the multilevel model (b-values). When significant interactions were found, stratified effect sizes were calculated in order to clarify group differences, using the STATA LINCOM command to calculate the appropriate linear combinations from the model containing the interaction.

#### Descriptives

ANOVAs (for continuous variables) and Chi-square (for categorical variables) tests were conducted to investigate group differences in demographic characteristics and ESM variables.

#### Emotional stress reactivity

To investigate whether stress (activity, event, social) elicited differential emotional reactions in the two groups, two multilevel analysis was conducted with (a) *NA* and (b) *PA* as the dependent variables and *group (low versus persistent)*, (*event, social, activity) stress* and their interaction as the independent variables [26].

#### Psychotic stress reactivity

To investigate whether stress (activity, event, social) elicited differential psychotic reactions in the two groups, a multilevel analysis was conducted with *paranoid ideation* as the dependent variable and *group (low versus persistent)*, (*event, social, activity) stress* and their interaction as the independent variables [28].

## Results

### Sample characteristics

The final sample included 529 participants, of whom 62 (12%) were in the persistent group and 467 (88% of the sample) in the low psychosis group, who completed a total of 21270 valid ESM observations (mean  = 38.40, SD  = 6.56). The groups did not differ in mean number of ESM reports (low group: mean (SD)  = 37.05 (6.92); persistent group: 36.58 (5.87); F = 0.26, p = 0.61). The two groups did not differ in age (F(1, 525) = 0.55, p = 0.46, marital status (χ^2^ (1)  = 0.06, p = 0.80, and educational level (χ^2^ (3) = 1.29, p = 0.73). Group differences on ESM variables are summarized in Table 1.

**Table 1:** Daily life descriptives by group.

	Low group	Persistent group	p-value
paranoia, mean (SD)	1.14 (0.28)	1.31 (0.50)	<0.001
negative affect, mean (SD)	1.26 (0.34)	1.50 (0.51)	<0.001
positive affect, mean (SD)	4.43 (0.87)	4.37 (0.79)	0.606
event stress, mean (SD)	0.23 (0.26)	0.25 (0.22)	0.475
activity stress, mean (SD)	2.58 (0.62)	2.63 (0.63)	0.549
social stress, mean (SD)	2.29 (0.72)	2.49 (0.73)	0.048

Note. For the daily life, experience sampling variables, which included multiple observations over time from each participant, an individual mean was first calculated over all reports; these values were then aggregated to obtain the group mean and SD.

### Emotional stress reactivity

**Figure 1:**
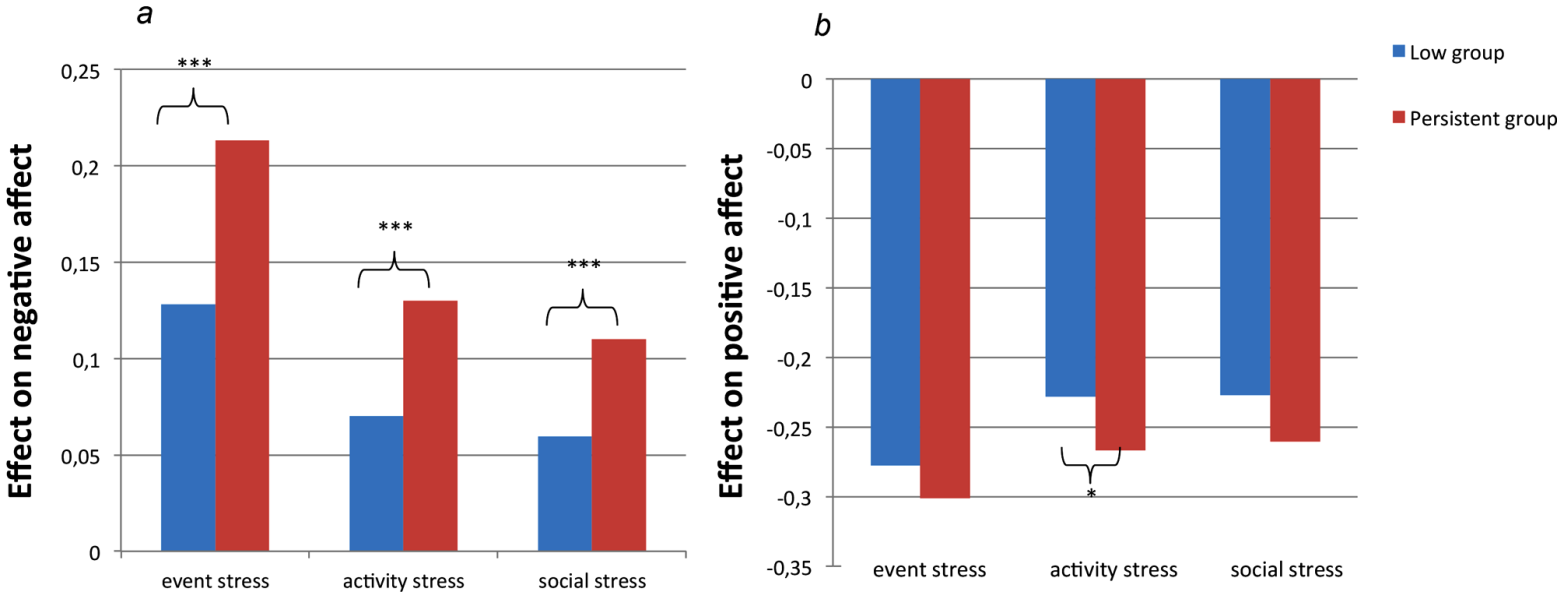
Emotional stress reactivity.

Multilevel linear regression analysis revealed significant interactions between group and all three stressors in the models predicting NA (event: b = .09; 95%CI .05, .12, p<0.001; χ^2^ (1)  = 21.54; activity: b = .06; 95%CI .04, .08, p = 0.000; χ^2^ (1)  = 42.4; p = 0.0000; social: b = .05; 95%CI .03, .07, p<0.001; χ^2^ (1)  = 21.49; p<0.001). For all stressors, stratified analyses showed a significantly greater increase in NA following stress in the Persistent group compared to the Low group (Figure 1).

Multilevel linear regression analysis revealed a significant interaction between group and activity stress in the model predicting PA (b = −.04; 95%CI −.07, −.01, p = 0.02; χ^2^ (1)  = 5.08), however not in the models with event stress and social stress predicting PA (event: b = −.02; 95%CI −.11, .007, p = 0.62; χ^2^ (1)  = 0.25; social: b = −.03; 95%CI −.07, .01, p = 0.10; χ^2^ (1)  = 2.65). For activity stress, stratified analyses showed a significantly stronger decrease in PA following stress in the Persistent compared to the Low group (Figure 1).

### Psychotic reactivity

**Figure 2:**
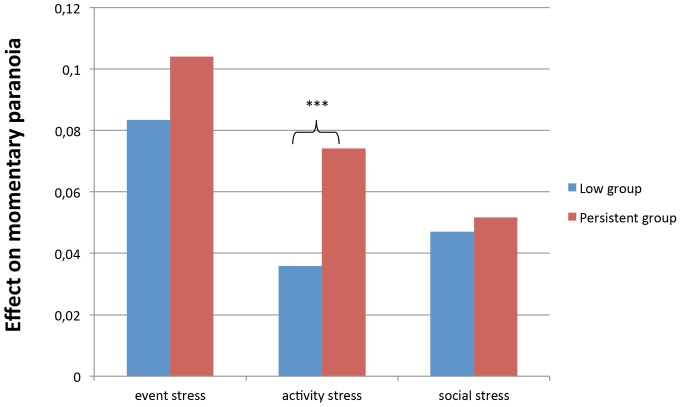
Psychotic reactivity to stress in the Persistent and the Low group.

Multilevel analyses revealed a significant interaction between activity stress and group in the model of momentary paranoid ideation *(*b = .04; 95%CI .02, .06, p<0.001; χ^2^ (1)  = 13.83). The Persistence group *(*b = .07; 95%CI .06, .09, p<0.001) reported significantly more momentary paranoid ideation when experiencing stressful activities compared to the Low group *(*b = .04; 95%CI .03, .04, p<0.001) (see Figure 2).

No significant interaction was apparent between group and event stress in the model of momentary paranoid ideation *(*b = .02; 95%CI −.02, .06, p = 0.33; χ^2^ (1)  = 0.95). Similarly, there was no significant interaction between group status and social stress in the model of momentary paranoid ideation *(*b = .00; 95%CI −.02, .03, p = 0.70; χ^2^ (1)  = 0.15).

### Sensitivity analyses – baseline psychopathology

Additional analyses were carried out, investigating whether baseline psychosis psychopathology influenced the results. All analyses were repeated with both exclusion of participants who reported delusions or hallucinations at baseline (low group n = 419 and persistent group n = 46 remained in the analyses) and controlling for baseline psychosis CAPE scores. Apart from some small effect size alterations, all results remained comparable (data not shown, available upon request).

## Discussion

The current study shows that sensitivity to daily life stress is associated with stable high levels of psychotic experiences over time. Although a causal relationship cannot be established, more stress reactivity may predict more persistence of psychosis over time. Thus, sensitivity at the micro-level is associated with persistence of psychotic experiences at the macro-level. More specifically, higher levels of emotional sensitivity (i.e. less expression of positive and more expression of negative affect in response to stress), and psychotic reactivity to daily stress were found in individuals with persistent subclinical psychotic experiences compared to individuals with persistently low levels of psychotic experiences.

**Figure 3:**
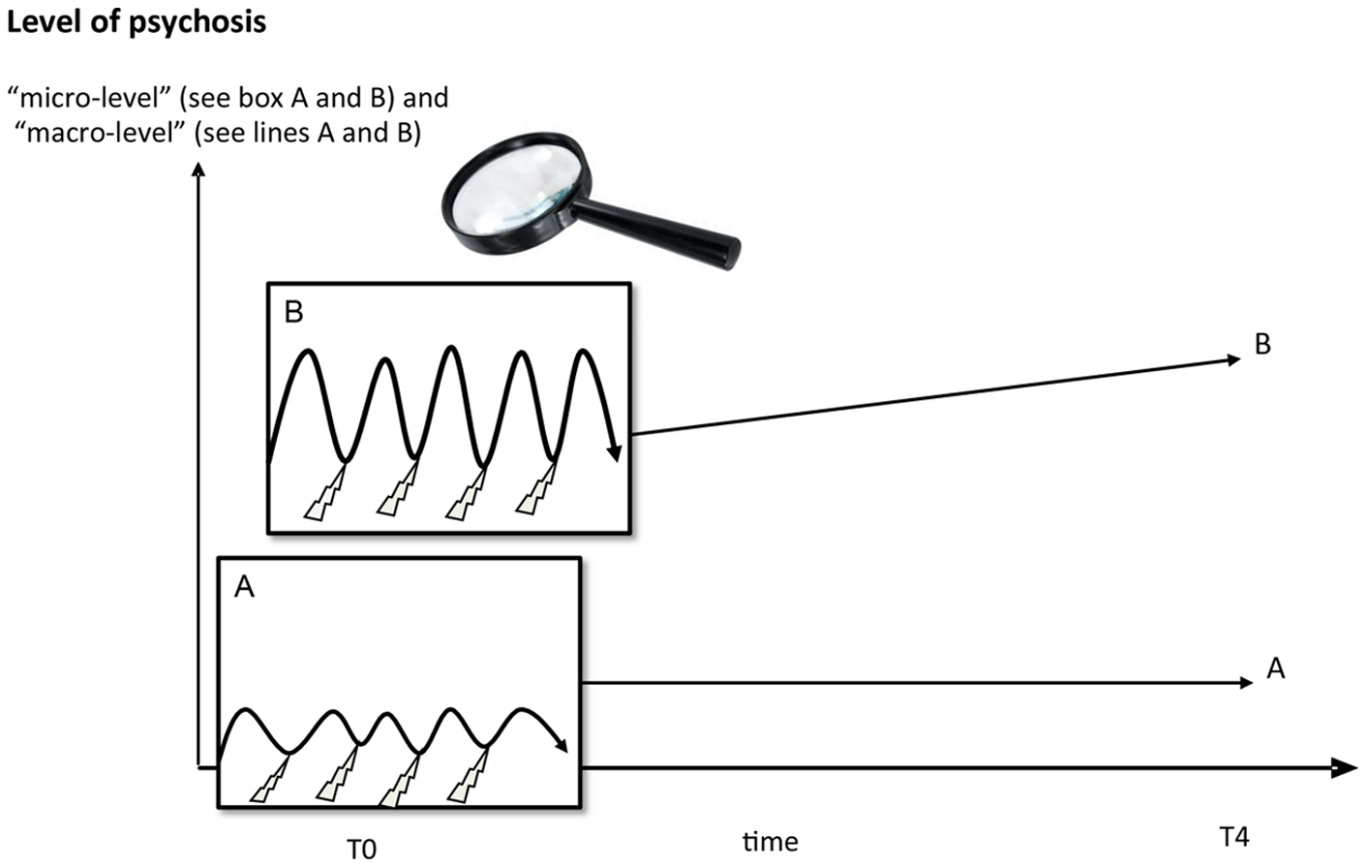
Behavioural phenotype of sensitisation at micro and macro level.

Individuals with persistent subclinical psychotic experiences were more emotionally responsive to all types of daily stressors (event, social and activity stress). This was more consistently found in models of NA reactivity compared to PA reactivity. Earlier ESM studies have found increased emotional stress reactivity in patients with psychotic disorders [13]. The current results extend these findings further to the subclinical expression of liability to psychosis at the level of the general population, i.e. before individuals develop any need for care. Higher levels of reactivity to stress in individuals with persistent psychotic experiences possibly may reflect early signs of sensitization. Thus, sensitization may be the mechanism underlying increased liability to develop, first, incidental and, second, persistent psychotic experiences, which in turn may be predictive of development of psychotic disorder [5]. The results suggest that the persistence of normally transient expression of psychotic experiences at macro-level may be traced back to baseline micro-level stress reactivity in daily life (Figure 3).

The findings indicate that individuals who are more responsive to stress are more likely to pertain to a group of individuals with persistently high levels of psychotic experiences. One important factor underlying these findings may be type and amount of coping. Thus, individuals who are liable to develop psychosis are more likely to (i) be more sensitive to stress [29] and to (ii) display dysfunctional coping [30] [31]. Given that coping is modifiable using the principles of cognitive behavioral therapy, future work may examine the relevance of coping in the earliest trajectories of psychosis expression [32]. Following from this, an important implication of our findings is the potential for (very) early recognition of risk and, possibly, risk reduction [15]. Strategies like these could bring about increased awareness of an individual's tendency of experiencing daily hassles unusually stressful, with the possibility of risk reduction preventing further progression of sensitization and a vicious cycle of increasing psychopathology.

An innovative aspect of the study is that it introduces a multilevel approach, linking different levels of manifestation of psychosis liability, namely “micro-level” daily life interactions and “macro-level” psycho(patho)logical experiences. This may represent an important step forward in complementing the current, diagnosis-oriented approach in psychiatry. Moreover, the results are in line with earlier propositions that liability to develop psychosis is distributed in the general population [1], and furthermore suggest that increased liability is reflected in part in heightened sensitivity in the form of emotional and psychotic response to environmental change. Taken together, the results suggest that epidemiology could significantly benefit from a stronger focus on daily life.

The results of the current study should be interpreted in the light of its strengths and limitations. First, use of ESM booklets instead of electronic devices means that the exact timing of participants' self-reports cannot be established with 100% certainty [33]. However, results of a study comparing self-reported and electronically monitored collection times, with the same intensive, semi-random time-sampling protocol used in the current study, indicated that self-reported collection times corresponded well with the electronic time-stamps [34]. Another comparative study concluded that paper and electronic diaries yield similar results [35]. Since both the ESM data collection and the first assessment of the CAPE were carried out at the same baseline measurement point, our data cannot be interpreted as showing that emotional/psychotic reactivity really “predicts” persistence. Rather, from the temporal perspective of the statistical model with baseline ESM data as dependent variable, we showed that those with stable high levels of psychotic experiences tended to be more emotionally and psychotically reactive to stress than those with stable low levels of psychotic experiences. The distinction between the groups is based on non-presence versus presence and/or persistence of presence of high levels of psychotic experiences. It cannot be ruled out that persistence of psychotic experiences was already present before the onset of the study, meaning that causality from daily life interactions to symptom persistence cannot be directly inferred. More prospective research is needed to unravel this causal chain over time. Moreover, it should be mentioned that in this sample we could only identify two developmental trajectories of psychotic symptoms; more dynamic developmental trajectories (i.e. increasing and/or decreasing), that were identified in earlier studies were not present. This may be due to the age range of the current sample, which was older than samples in earlier studies. Therefore, dynamic changes in psychotic symptoms may already have been stabilized. It should moreover be mentioned, that only one ESM item was used to measure subclinical daily life psychotic experiences. However, as this study constitutes a general population study, ESM items were chosen to capture subclinical psychotic phenomena with enough momentary variation. The ESM item referring to paranoia was the only ESM item on psychotic phenomena that was included in the study. The current study also had some specific strengths. In particular, the repeated sampling of daily experiences and interactions over 5 days takes into account daily variation of psychological phenomena, providing an ecologically valid picture of a person's psychological functioning. Multiple measures per person were complemented by a relatively large number of participants. Use of multilevel modeling allowed assessment of within-person associations between stress and subjective experiences in real time and real-life contexts. Such intensive sampling offers the possibility to detect subtle deviation of emotional or psychotic reactivity at the micro-level that might easily be missed when testing at the macro-level and that may be especially important in the subclinical ranges of the extended psychosis continuum, when the very first expression of (liability for) psychosis may become manifest. The approach of the current study offers an exciting angle to link different levels of manifestation of psychosis liability; future research should focus on replication of the findings and on their extension to other ranges of the extended psychosis continuum.
